# Systematic Evaluation of HILIC Stationary Phases for Global Metabolomics of Human Plasma

**DOI:** 10.3390/metabo12020165

**Published:** 2022-02-09

**Authors:** Farideh Hosseinkhani, Luojiao Huang, Anne-Charlotte Dubbelman, Faisa Guled, Amy C. Harms, Thomas Hankemeier

**Affiliations:** Leiden Academic Centre for Drug Research, Division of Systems Biomedicine and Pharmacology, Leiden University, 2300 RA Leiden, The Netherlands; f.hosseinkhani@lacdr.leidenuniv.nl (F.H.); l.huang@lacdr.leidenuniv.nl (L.H.); a.c.dubbelman@gmail.com (A.-C.D.); f.guled@lacdr.leidenuniv.nl (F.G.); a.c.harms@lacdr.leidenuniv.nl (A.C.H.)

**Keywords:** HILIC, untargeted metabolomics, polar metabolites, plasma metabolomics, liquid chromatography

## Abstract

Polar hydrophilic metabolites have been identified as important actors in many biochemical pathways. Despite continuous improvement and refinement of hydrophilic interaction liquid chromatography (HILIC) platforms, its application in global polar metabolomics has been underutilized. In this study, we aimed to systematically evaluate polar stationary phases for untargeted metabolomics by using HILIC columns (neutral and zwitterionic) that have been exploited widely in targeted approaches. To do so, high-resolution mass spectrometry was applied to thoroughly investigate selectivity, repeatability and matrix effect at three pH conditions for 9 classes of polar compounds using 54 authentic standards and plasma matrix. The column performance for utilization in untargeted metabolomics was assessed using plasma samples with diverse phenotypes. Our results indicate that the ZIC-c HILIC column operated at neutral pH exhibited several advantages, including superior performance for different classes of compounds, better isomer separation, repeatability and high metabolic coverage. Regardless of the column type, the retention of inorganic ions in plasma leads to extensive adduct formation and co-elution with analytes, which results in ion-suppression as part of the overall plasma matrix effect. In ZIC-c HILIC, the sodium chloride ion effect was particularly observed for amino acids and amine classes. Successful performance of HILIC for separation of plasma samples with different phenotypes highlights this mode of separation as a valuable approach in global profiling of plasma sample and discovering the metabolic changes associated with health and disease.

## 1. Introduction

Metabolomics is rapidly becoming a powerful approach in its application to system biology-based studies. It enables characterizing the profile of small molecules in biological samples and thereby discovering metabolites that discriminate across phenotypes [1,2]. In-depth investigations of metabolic pathways highlighted polar and ionic compounds such as amino acids, carboxylic acids, phosphorylated compounds, nucleotides, sugars etc., as key modulators of physiological and pathophysiological processes [3,4]. Metabolomics studies are typically performed using liquid chromatography (LC) hyphenated to mass spectrometry (MS), and reversed-phase (RP) LC is the most widespread choice in bioanalytical separation as it generates reproducible data for a large set of metabolites. However, the analysis of polar and ionic compounds continues to challenge this mode of chromatography as they are poorly retained on the stationary phase of RPLC columns [5]. Even though chemical derivatization of these compounds is an active research area, disadvantages of this approach include partial derivatization and increased sample complexity. Moreover, tedious derivatization steps make it a time-consuming method, and additional experimental steps may influence the recovery rate, accuracy and repeatability of the result [6]. To that end, hydrophilic interaction liquid chromatography (HILIC) has grown in popularity for the chromatographic retention and separation of polar compounds [7]. In HILIC, the high organic content mobile phase prolongs retention time, and improves mass spectral detection of polar metabolites by increasing the ionization efficiency [8]. The separation mechanism includes a combination of various hydrophilic, electrostatic, and ionic interactions; however, the dominant mechanism depends on the physicochemical property of the stationary phase (neutral, charged and zwitterionic) and the structure of the analytes [7]. Among all commercially available stationary phases, the neutral stationary phase of Acquity-Waters (UPLC BEH-amide) and the zwitterionic (ZIC) stationary phases of SeQuant^®^ Merck (HPLC Phosphorylcholine and Sulfobetaine) have often been exploited for the separation and targeted analysis of polar metabolites. For instance, BEH-amide has recently been utilized for effective separation of 24 amino acids from cell culture [9] and could successfully cover key metabolite classes such as sugars, amino acids, nucleotides and organic acids in plasma samples [10]. Arase et al. [11] reported the phosphorylcholine type ZIC-cHILIC as the most appropriate column for the separation of nucleotides. In another study, ZIC-HILIC has been applied for determination of organic acids in plasma and urine for the assessment of acidosis in patients with severe malaria [12].

Despite the increased interest in HILIC in the last decade for targeted profiling of polar metabolites, the potential of HILIC in untargeted metabolomics is underestimated due to low reproducibility, complex retention mechanisms and low peak capacities [13]. Even though several comparison studies investigated the metabolic coverage and retention mechanisms involved in different HILIC columns, the lack of a practically helpful guide has resulted in no consensus on the utilization of the appropriate column and analytical procedure. In a recent study, Contrepois et al. [14] reported that, at neutral pH, a superior separation and higher metabolic coverage in plasma and urine were achieved by ZIC-HILIC when testing five columns, including BEH-amide and BEH-HILIC (charged). In another study, Sillner et al. [15] observed better performance of BEH-amide at basic pH compared to a zwitterionic column in terms of peak width, isomers separation, and the number of detected features in fecal samples. 

In addition to the metabolic coverage, matrix effect and batch repeatability are crucial factors that need to be considered in untargeted metabolomics studies of biological samples. Inorganic ions such as sodium, potassium and chloride are among the major electrolytes in different body fluids. These ions are well known to affect electrospray ionization by promoting adduct ions with sample analyte and cluster ion formation with mobile phase additives, and leading to ion suppression [16]. The use of RP-LC often diminishes these problems as salts generally elute with the void volume. However, when employing HILIC, these inorganic ions can be retained by the chromatographic system and impact the detection of coeluting polar analytes [17]. Most previous studies have only focused on the effect of buffer salt on the retention mechanism [18,19], while far too little attention has been paid to the matrix salt effect on the analysis of diverse polar metabolites. 

This study set out to systematically explore the selectivity of HILIC interaction and coverage for two popular HILIC columns; BEH-amide and ZIC-c HILIC. We scored the performance of two stationary phases (zwitterionic and neutral) for nine different classes of compounds at three different pH conditions (acidic, neutral, basic). Parameters were evaluated in terms of number of detected metabolites, peak shape, retention factor, resolution and sensitivity. To assess the intra- and inter-batch column repeatability in measuring biological samples, we monitored the peak area variation and retention time shift using plasma samples. Plasma matrix effect, particularly salt effect on neutral and zwitterionic stationary phases, was also evaluated. Finally, we assessed the column performance for utilization in untargeted metabolomics using plasma samples with diverse phenotypes. Comparison based on the total feature numbers and retention distribution of detected features was carried out to determine the optimal HILIC stationary type. Since plasma matrix contains different polar metabolites, many of which elute close to the solvent front in RP chromatography, HILIC separation can enhance plasma metabolome coverage and provide an enhanced view on plasma composition.

## 2. Results and Discussion

### 2.1. Column Selectivity and Performance

Two prominent HILIC columns (BEH-amide and ZIC-c) were tested for selectivity and performance for nine classes of compounds comprising 54 polar metabolites (Table S1). Previous studies [14] often utilized the same separation gradient while comparing different HILIC columns. However, the different structure of stationary phases might require different chromatography conditions for obtaining a good performance [20]. Therefore, we used a specified gradient for each column that could retain most of the tested analytes. We aimed at using a scoring approach that allows to compare the performance of different chromatographic conditions for the analytes of interest based on the following parameters: metabolite retention, peak sensitivity, peak sharpness and peak symmetry [14,21]. The metabolite performance was considered as good, acceptable, or bad, based on the calculated total score (see Table S4, supporting information). Metabolites scored as “good” exhibit long retention (void time × 4) and avoid the ion suppression zone. In addition, their narrow peak profile together with minimal tailing and high sensitivity provide more reliable metabolite identification and quantification. Metabolites evaluated as “acceptable” can be used for qualitative purposes but might introduce problems during quantitative analysis. Metabolites evaluated as “bad” are not recommended to be measured using the corresponding HILIC method due to either broad peak profile, early elution or low peak intensity [14].

**Figure 1:**
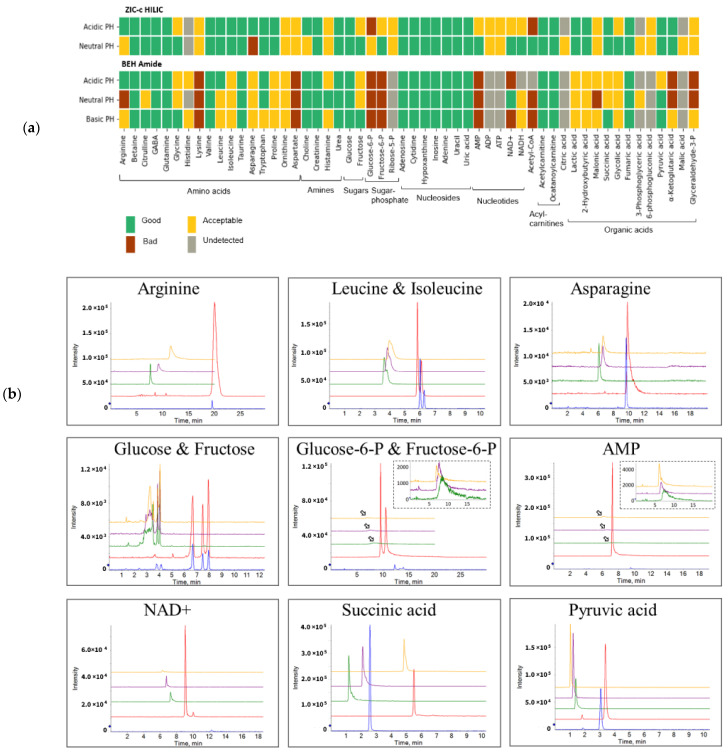
Overview of performance score and selectivity under chromatographic conditions. (**a**) Individual score of column performance for polar metabolites from different classes of compounds. (**b**) Column selectivity and chromatographic peak performance of representative metabolites from different classes under five different LC method conditions. Anomer mutarotation causes the glucose signal to split into two separate peaks as shown on both columns. The arrows represent the zoom figures. Blue: ZIC-c HILIC at pH 3; Red: ZIC-c HILIC at pH 7; Green: BEH Amide at pH 3; Purple: BEH Amide at pH 7; Yellow: BEH Amide at pH 10.

Figure 1a shows the performance cumulative score per chromatographic condition for each individual metabolite. The ZIC-c column operated at neutral pH was superior in coverage and peak performance, as the majority (96%) of tested standards achieved a good or acceptable performance score. In comparison, the BEH-amide showed the best performance at basic condition with 74% of metabolites obtaining good or acceptable score. From the standard working solution (20 μM), most compounds were detected by ZIC-c at both acidic and neutral pH, except for citric acid, glycerate-3-phosphate and malic acid, which were only detected under the neutral condition. In contrast, BEH-amide resulted in bad performance for the above-mentioned organic acids, as well as for 6-phosphogluconic acid, ribose-5-phosphate, ADP and ATP. Regardless of the pH, both columns exhibited good chromatographic performance for amines, nucleosides and acyl carnitines, but sugar phosphates, nucleotides and coenzyme-A only performed well on ZIC-c. 

Figure 1b presents the column selectivity and chromatographic peak performance of representative metabolites from different classes under the five different LC method conditions. Baseline separation of the isomers, leucine and isoleucine, fructose and glucose, glucose 6-phosphate and fructose 6-phosphate could be achieved with the ZIC-c column only. 

This finding is consistent with that of Contrepois et al. [14] who reported a superior separation and higher metabolic feature coverage in plasma and urine using a zwitterionic column (ZIC-HILIC) at neutral pH among five tested columns, including BEH-amide and BEH HILIC (charged). Similarly, among six different HILIC stationary phases, Tufi et al. [22] proposed ZIC-c for simultaneous analysis of neurotransmitters in cell extracts. In fecal samples, Sillner et al. [15] also compared the performance of silica, amide, and zwitterionic columns for fecal polar metabolites and concluded that the zwitterionic column (ZIC-c) provided superior coverage and selectivity.

### 2.2. Retention Mechanism

**Figure 2:**
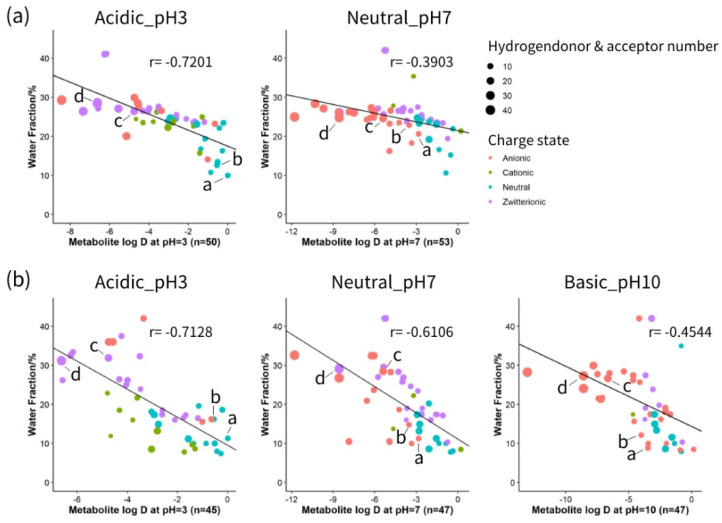
Analysis of the relative contribution to the retention mechanism on different HILIC columns. (**a**) Correlation analysis on ZIC-cHILIC and (**b**) Correlation analysis on BEH-amide. Individual metabolites are shown as a: 2-hydroxybutyric acid; b: lactic acid; c: AMP; d: NAD+.

HILIC separation is generally regarded as a mixed-mode mechanism. Partitioning of solutes occurs between the surface aqueous layer and the relatively non-polar organic mobile phase based on the polarity of the solutes. Electrostatic attractions or repulsions are created between charged solutes and the stationary phase. In addition, molecules with hydrogen-donor or hydrogen-acceptor groups can interact through the hydrogen bonds with the stationary phase [23]. To gain a better insight into the interaction of metabolites with zwitterionic and neutral stationary phases under different pH values, correlation analysis was performed between the physical and chemical properties of a variety of compounds and their retention on each column. Physical and chemical properties including logD value, charged state and hydrogen bonding of 54 metabolites under acidic, neutral and basic pH are listed in Table S3. As depicted in Figure 2, a linear relationship was observed between metabolites polarity (as indicated by logD) at different pH conditions and elution order (as shown by the percentage of water phase). The impact of charge state is displayed as different dot colours, and the size of the dot reflects the influence of hydrogen bonding. At pH 3, both columns showed a relatively high correlation between logD and water fraction, which indicates that under acidic conditions, the separation mechanism is mainly governed by the partitioning of the analytes between the organic-rich bulk and water-rich layer. However, as pH increases, the correlation factor between logD and water fraction decreases. Increasing the pH from 3 to 7 converts the charge of organic acids from neutral to anionic, and nucleotides from zwitterionic to anionic (Table S3). Therefore, in the ZIC-c column at pH 7, anionic compounds such as 2-hydroxybutyric acid and lactic acid provoke more electrostatic attraction with the positively charged quaternary ammonium part of the phosphorylcholine functional group, indicating that a higher percentage of water is needed for elution. However, this effect was minimal for nucleotides such as AMP and NAD+ that required a higher percentage of water for elution in pH 3 as well. This can be explained by the fact that although these compounds are zwitterionic at pH 3 and demonstrate no net charge, they are still able to participate in electrostatic interactions with zwitterionic functional groups of the column. 

Compared to ZIC-c, BEH-amide shows relatively higher involvement of partitioning at neutral pH with a higher correlation coefficient. However, at basic pH, partitioning is decreased by electrostatic interactions due to ionization of residual silanol groups, that causes even less retention or exclusion of anionic compounds [8,13]. In terms of polar interactions, hydrogen bonding also influences compound retention on both BEH-amide and ZIC-c HILIC columns. For each charge group, it was observed that compounds with higher total hydrogen donors and acceptors retain longer on the column. In line with previous studies [23,24], our results also demonstrated the mixed mode of retention mechanisms in neutral and zwitterionic HILIC. Hydrophilic partitioning interaction plays a primary role in separation. In addition, both BEH-amide and ZIC-c columns exhibited hydrogen-bonding interaction, whereas ZIC-c showed stronger electrostatic interactions than BEH-amide.

### 2.3. Matrix Effect

**Figure 3:**
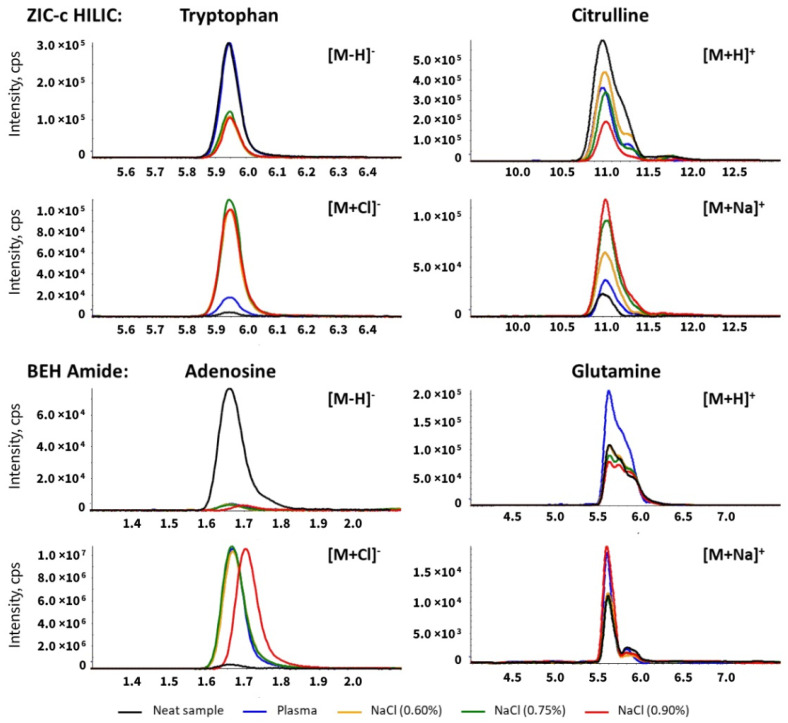
Extracted ion chromatogram of metabolites influenced by sodium chloride on the ZIC-c HILIC and BEH-amide columns. Citrulline and glutamine showed the increased MS responses of sodium adducts in plasma and salt sample (pure sodium chloride solution) versus neat sample (no sodium chloride). Tryptophan and adenosine showed the increased MS responses of chloride adducts in plasma and salt sample (pure sodium chloride solution) versus neat sample (no sodium chloride).

Inorganic ions such as sodium (Na^+^) and chloride (Cl^−^) are undeniably part of the plasma matrix. These ions are known to interfere with the electrospray ionization and affect the sensitivity in mass spectrometry [25]. This effect can be incremental in HILIC separation since these ions can be retained by the polar stationary phase and interact with the mobile phase additive [26]. To investigate the effect of matrix inorganic ions in untargeted HILIC-MS analysis, chromatographic separation and ionization of analytes of interest were compared with sodium chloride solution spiked with the standard mix. Blood plasma contains approximately 135–145 mmol/L sodium while the amount in 0.9% saline solution is 154 mmol/L. In order to closely mimic the plasma salt composition, we used sodium chloride solution at three concentrations: 0.6% and 0.75% and 0.9%. A major elution peak of sodium cluster series with the mobile phase component, formate, was detected as [Na(n+1) + HCOOn]^+^ in the positive mode and [Na(n) + HCOO(n+1)]^−^ in the negative mode around the highest water fraction (40%) during the HILIC gradient for both columns (Figure S2). Therefore, the coeluting metabolites arginine and lysine are directly influenced due to competition with the sodium cluster (Tables S5 and S6). Along with the increasing level of sodium chloride in samples, an increased signal of the [M + Na]^+^ adduct was observed on citrulline, glutamine (eluting around 10 min) and adenosine, ocatanoyl-carnitine, creatinine, which eluted at around 4mins on the ZIC-c column. Similarly, a slight increased [M + Na]^+^ adduct signal was detected for glutamine on the BEH-amide column at around 6mins (Table S7, Figure 3). No chloride ion cluster was directly detected, but a significantly increased adduct signal of [M + Cl]^−^ was detected for the indicated analytes in the sodium chloride sample on the ZIC-c column at around 5.7–6.0, 10 min, and on the BEH-amide column at around 1.45–1.8, 2.4–2.9 min (Table S7, Figure 3). Most compounds that eluted around these periods exhibited ion suppression due to adduct formation (Tables S5 and S6). 

In addition to the matrix effect caused by inorganic ions, the overall effect of plasma matrix on ionization was investigated as well. Table 1 summarizes the statistics for the entire plasma matrix and sodium chloride effect on representative metabolite classes. A comparable plasma matrix effect was observed on both columns, with 73% of 51 representative metabolites influenced on ZIC-c, and 68% of 47 representative metabolites influenced on BEH-amide. ZIC-c showed more susceptibility to the effect of sodium chloride than BEH-amide regarding the total number of affected metabolites as well as the measurement precision as shown in Tables S5 and S6. In particular, amino acids and amines showed more numbers affected by the presence of sodium chloride on ZIC-c. This significant ion suppression or enhancement caused by sodium chloride might be attributed to the constant competition between Na^+^, Cl^−^ and the zwitterionic amino acids, positively charged amines during the electrostatic interaction at pH 7 condition (Table S3).

For both columns, there was no significant increase in matrix effect value with increased sodium chloride concentration. Ion suppression with low matrix effect value was observed only in 0.9% group for a few metabolites. However, this still suggests that high-salt matrices can have a significant impact. Existing research attempted depletion of salt during sample preparation by methods such as cation and anion solid phase extraction, thereby enhancing the sensitivity [15]. However, repeatability and coverage of such methods needs more validation for biological samples with high concentrations of various salts. Overall, our results indicated that regardless of the column type, inorganic ions have affinity for the polar stationary phase in HILIC chromatography. Further investigation on depletion of these ions during sample preparation while having a high metabolic coverage is needed.

### 2.4. Repeatability Evaluation

**Figure 4:**
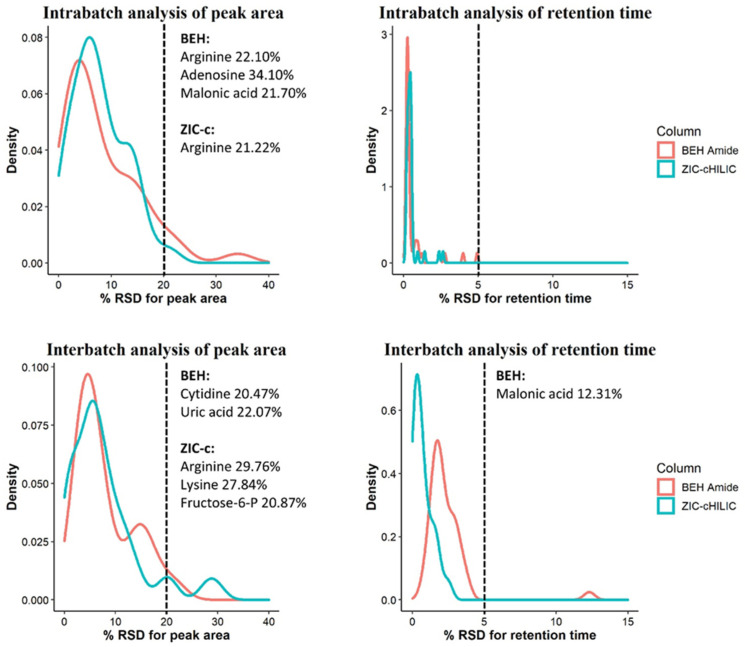
Repeatability evaluation of peak areas and retention times of 42 representative compounds during inter- and intra-batch analysis using ZIC-c HILIC and BEH-amide columns. Metabolite names with an RSD above 20% are listed on the right side of the cut-off line.

A key prerequisite for obtaining good metabolic profiles is repeatability. In terms of this, HILIC is recognized to be more challenging in long batch analysis than in RP columns due to the complex retention mechanism. Therefore, we further evaluated two HILIC columns for intra- and inter-batch repeatability using retention time (RT) and peak area stability of 42 representative polar compounds in a pooled plasma QC sample. The detailed results are summarized in Table S8, and supplemented by Figures S4 and S5. The distribution of relative standard deviations (RSD) for RT and peak area in intra- and inter-batch analysis are visualized in Figure 4.

#### 2.4.1. Intra-Batch Repeatability

RSD values below 30% for peak area define a high-quality dataset for untargeted analysis and reflects good method stability over runs with plasma matrix [27]. The intra-batch peak area repeatability of both columns was excellent; of 42 tested analytes, all showed an RSD < 20% on the ZIC-c HILIC except for arginine (21%), while for BEH-amide, all metabolites showed an RSD < 20% with exception of arginine (22%), adenosine (34%) and malonic acid (22%). Both columns also exhibited a good RT stability for all tested analytes with variation < 5% RSD, within 200 consecutive injections of pooled plasma QC sample.

#### 2.4.2. Inter-Batch Repeatability

Similarly, the inter-batch analysis revealed high RT stability in both columns with RSD < 5% for most of the analytes, with the exception of malonic acid (12%) on BEH-amide. The peak area analysis evaluations also showed satisfactory repeatability; on the ZIC-c HILIC, all metabolites showed an RSD below 20%, with the exception of arginine, lysine, fructose-6-P with relatively high RSD, ranging from 20% to 30%. On the BEH-amide, 40 metabolites showed an RSD below 20% while this value was relatively high for cytidine and uric acid ranging from 20% to 35%. 

Although we performed a pre-batch run with 20 QC plasma samples on both HILIC columns, malonic acid showed improved peak shape after more sample injections on BEH-amide (Figure S3). Similarly, increased peak responses were observed for arginine, lysine, fructose-6-P, and adenosine. In contrast, decreased peak responses were observed for cytidine and uric acid on BEH-amide. The accumulated matrix that remained in the ion source might cause changes in the peak response as well. This can be alleviated through MS ion source cleaning between batches. Nucleosides eluting in the early stage of the gradient on the BEH-amide column showed less peak area repeatability than on the ZIC-c HILIC column. Although the chromatographic repeatability has been considered a common challenge in HILIC technology, our results demonstrate that the deviation of peak areas and retention times across inter- and intra-batch analysis did not vary significantly, which reflects good method stability and enables reliable chromatogram alignment and peak matching across different samples.

### 2.5. Untargeted Metabolomics Analysis for Human Plasma

**Figure 5:**
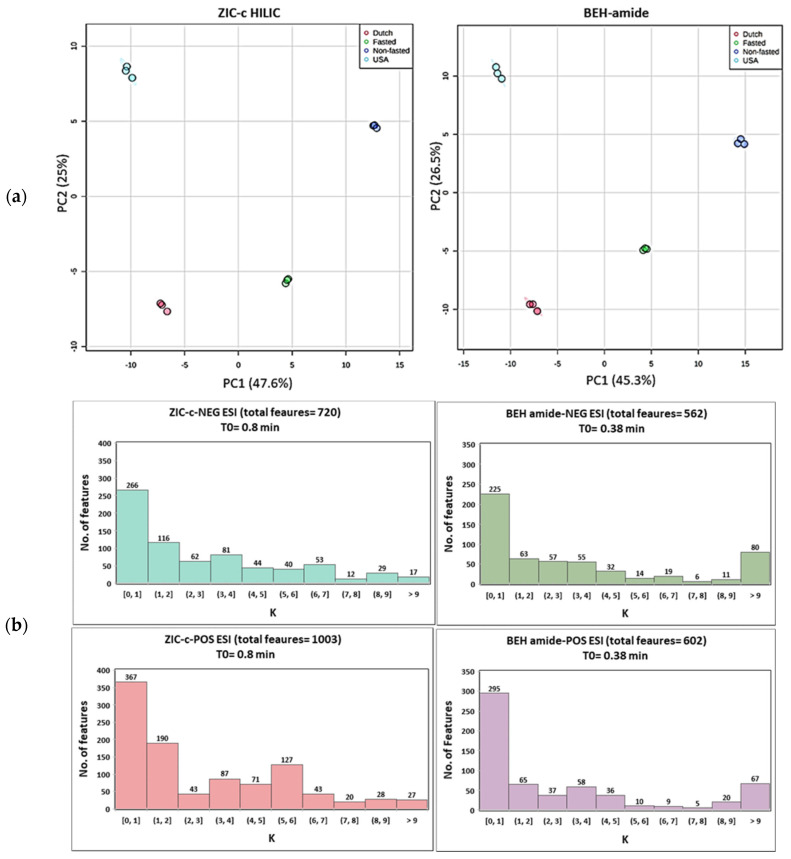
(**a**) Principal component analysis (PCA) score plot of pre-processed untargeted features in ZIC-c and BEH-amide. Each phenotype was subjected to a triplicate sample analysis; (**b**) Retention factor (K) distribution of detected features on each column.

Untargeted metabolomics analysis of plasma samples from four different phenotypes were utilized to estimate and compare the metabolic coverage of each column. The quality of generated chromatographic peaks has a great effect on the coverage output. When using HILIC for global metabolomics, one drawback is the presence of poor chromatographic peaks (broad peaks, multiple peaks and tailing peaks). The total ion chromatograms of the plasma samples and some examples of poor peak shapes are presented in supplementary Figures S6 and S7. During data processing of this type of peaks, many features with the same *m*/*z* could be generated by the peak extraction algorithm while multiple features are generated by a single metabolite. Therefore, following untargeted HILIC-MS analysis, data were extensively pre-processed using XCMS, MS-FLO and in-house tools, followed by strict rules of feature removal to omit isotopes, background peaks (in procedure blank per method), and also unreliable retention areas (which restricted the inclusion of peaks with broad tailing). The different steps of the data pre-processing and the feature yield per step are summarized in Table S9. Two-dimensional PCA score plots (Figure 5a) revealed a visible separation in metabolic profiles induced by different plasma phenotypes (Dutch, USA, fasted and non-fasted) on both HILIC columns. The first two principal components, respectively, explain 47.6% and 25.0% of the total variance of the data for ZIC-c HILIC, while those for BEH-amide explain 45.3% and 26.5%, respectively. In addition to the successful separation, replicates of each phenotype are clustered together in the plot showing good repeatability in the HILIC-MS. The lower intra-group variation on BEH-amide can be explained by the lower number of covered features. In total, 720 and 1003 *m*/*z* features were detected on the ZIC-c column, respectively, under negative and positive ESI modes, while a total of 562 and 602 *m*/*z* features were detected on the BEH-amide column using negative and positive ESI modes, respectively. In addition to the higher coverage, ZIC-c HILIC was more successful in isomer separation which is another necessary evaluation in LC condition optimization for untargeted metabolite profiling. For example, among the negative and positive features, 18 and 39 pairs of putative isomers were detected, respectively, on ZIC-c column, while 10 and 13 pairs were detected, respectively, on BEH-amide column. The successful isomer separation by ZIC-c might be attributed to the selectivity of the positively charged stationary phase for acidic isomers, which are negatively charged at neutral pH. 

Investigation of the column retention mechanisms revealed partitioning as a major retention mechanism in both ZIC-c and BEH-amide columns. This may have resulted in an overall similar retention distribution of plasma features detected in the untargeted analysis in both columns (Figure 5b). Features eluting in the early volume zone are represented in K [0, 1]; they accounted for the highest percentage in both columns, with an average 44.5% of total features on BEH-amide, compared to an average of 36.8% on ZIC-c HILIC. In contrast, the number of features with longer retentions, represented in K ≥ 1, obviously decreases. Due to the presence of strong electrostatic interactions in ZIC-c HILIC, more balanced retention profiles were observed in the range of K ≥ 2, which is important for untargeted analysis to reduce co-elution and selectively cover more features.

## 3. Materials and Methods

### 3.1. Chemicals and Materials

Analytical grade solvents acetonitrile, methanol, chloroform and formic acid (98%) were purchased from Biosolve (Biosolve BV, Valkenswaard, The Netherlands), whereas ammonium formate (≥99.995%), ammonium hydroxide (28−30 wt % solution of ammonia in water) sodium chloride (≥99.0%) and sodium hydroxide were obtained from Sigma Aldrich (Sigma-Aldrich, Burlington, WV, USA). MilliQ water was obtained from a Merck Milli-pore A10 purification system (Raleigh, NC, USA). Chemical standards and stable isotope-labelled standards were purchased from Sigma-Aldrich (St. Louis, MO, USA) unless otherwise mentioned. The complete information of the (stable isotope-labelled) standards and suppliers are provided in the supporting information Table S1. An EDTA pooled plasma (June 2020) was used for the column performance, matrix effect, and repeatability evaluation and purchased from Innovative Research (Peary Court Novi, MI, USA). Four diverse EDTA plasma samples (categorized as Dutch, American, fasted and non-fasted) were used for untargeted analysis. American, fasted and non-fasted pooled plasmas were purchased from Innovative Research (Peary Court Novi, MI, USA) and Dutch pooled plasma was purchased from Sanquin (Sanquin, Amsterdam, The Netherlands).

### 3.2. Standard Solutions

A total number of 54 authentic standards were used during the experiment covering a wide range of polar metabolic classes including amino acids (17), amines (4), sugars (2), sugar phosphates (3), nucleosides (7), nucleotides (5), acyl-carnitines (2), coenzyme A (1) and organic acids (13). Stock solutions of the analytes were prepared at a concentration of 10mM in milliQ water, except for aspartic acid, adenosine, adenine, uracil which were prepared in methanol and water (1:1, *v*/*v*). For certain standards, addition of 0.05% formic acid (aspartic acid, adenosine, adenine, uracil) or 0.1 M sodium hydroxide (hypoxanthine, uric acid) was needed to assist dissolution. Stock solutions were stored in Eppendorf tubes at −80 °C. A standard mix solution was prepared by mixing 54 individual standard solutions at a final concentration of 160 μM. The standard mix solution was aliquoted in separate Eppendorf tubes and also stored at −80 °C. Standard working solutions were prepared at a final concentration of 20 μM by dilution of the standard mix solution in acetonitrile/milliQ water (9:1, *v*/*v*). 

Stable isotope-labelled standards (SILs) used in the study include U-13C4, U-D3, 9-15N-aspartate, U-13C5-glutamine, 2,3,3-D3-leucine, D4-choline, U-13C6-glucose, U-15N2-UMP, D3-carnitine, 13C3-pyruvate, 2,2,3,3-D4-succinate, 2,2-D2-glycine, 2,3-D2-fumarate, U-13C11, U-15N2-tryptophan, U-13C4, U-15N2-asparagine, U-13C5, U-D5, 15N-glutamate, U-13C5-valine, U-13C6-lysine and U-15N2-UMP. The SILs stock solution was prepared at concentration of 10 mM in milliQ water and stored at −80 °C. Together with the quality control sample, this mix was used for day-to-day analysis to monitor the process and analytical variation.

### 3.3. Sample Preparation

Plasma samples were extracted using chloroform/MeOH/water solvent system (2.6/2.0/2.4, *v*/*v*/*v*) according to Sostare et al. [19]. Briefly, 100 μL of plasma was quenched using 75% ice-cold methanol (400 μL MeOH + 132 μL milliQ water) and 200 μL of chloroform. The mixture was subsequently vortexed for 3 min and centrifuged for 10 min (13,000× *g*, 4 °C). The upper layer of supernatant (~600 μL) was transferred into a new Eppendorf tube and then mixed with 200 μL of chloroform and 230 μL of milliQ water. The mixture was vortex mixed for 3 min and centrifuged for 10min (13,000× *g*, 4 °C) for a second liquid-liquid extraction (LLE). The final upper layer (~900 μL) was collected and evaporated to dryness in a Labcono SpeedVac (Labcono, Kansas City, MO, USA). The residue was then reconstituted with 100 μL of acetonitrile/ milliQ water (9:1, *v*/*v*). In order to increase the quenching and minimize the residual enzymatic activity, all solvents for extraction in this study were used ice-cold. To improve the sensitivity for untargeted analysis, those plasma samples were processed in the same manner only reconstituted in 50 μL of reconstitution solution.

A Quality control (QC) sample was prepared by pooling 50 μL of every study sample, spiked with standard mixture solution and SILs solution at a final concentration of 20 and 40 μM, respectively. This QC sample was used for monitoring the stability, precision, random errors and correcting for instrument fluctuations during the analytical run.

### 3.4. Instrumentation and LC-MS Acquisitions

HILIC chromatography was performed using a Waters Acquity UPLC Class II (Waters Chromatography Europe BV, Etten-Leur, The Netherlands) with the oven temperature set at 30 °C. Two HILIC columns were investigated in this study, the Acquity BEH-amide column (2.1 mm × 100 mm, 1.7 μm, Waters, Irland) and the SeQuant^®^ ZIC^®^-cHILIC column (2.1 mm × 100 mm, 3.0 μm- Merck, Darmstadt, Germany). The mobile phase composition was the same for both columns. Mobile phase A consisted of 90% acetonitrile and 10% 5 mM ammonium formate. Mobile phase B consisted of 10% acetonitrile, 90% 5 mM ammonium formate. The acidic and basic pH of the aqueous 5 mM ammonium formate were adjusted using formic acid and ammonium hydroxide, respectively. The BEH-amide column is composed of neutral ethylene bridge hybrid (BEH) particles which enables the stability of the column over a wide range of pH (2–11). Therefore, BEH-amide column was operated at 3 different pH values of mobile phase; acidic (pH 3), neutral (pH 7) and basic (pH 10). The ZIC-c column is composed of silica-based particles (1:1 charge-balanced phosphorylcholine functional group) which lose stability in pH above 8. Therefore, the ZIC-c was operated only at acidic and neutral conditions.

For each HILIC column, the gradient and flow rate were optimized based on the standards retention (Table S2). The flow rate used with SeQuant^®^ ZIC^®^-cHILIC column was 0.25 mL/min and the starting gradient condition was 0% B for 2 min, changing linearly to 40% B over the next 20 min, after which the solvent composition returned to starting condition over 0.1 min, followed by re-equilibration for 10 min prior to the next injection. The flow rate applied on Acquity BEH-amide column was 0.5 mL/min and the starting gradient condition was 0% B for 1.2 min, changing linearly to 40% B over the next 14 min, ends up with 0% B from 14.2–18 min. 

Mass spectrometry experiments were carried out on a quadrupole-TOF (SCIEX 5600 TripleTOF, AB SCIEX, Foster City, CA, USA). Electrospray ionization (ESI) was operating at both positive and negative ion mode. The ESI source parameters were as follows (positive/negative ion mode): spray voltage ±4.5 kV, capillary temperature 550 °C, sheath gas 50, auxiliary gas 65, curtain gas 25. The full scan mode was applied for data acquisition over a *m*/*z* range of 50–900 Da. The complete compound list with observed mass adducts and *m*/*z* ratios is shown in Table S1. In total, five LC conditions were analysed with both positive and negative ESI TOF-MS.

### 3.5. Matrix Effect

Matrix effect (i.e., suppression and enhancement of metabolite signal in [M − H]^−^ or [M + H]^+^) was evaluated by the post extraction addition method which is based on a quantitative signal comparison between a plasma matrix spiked with standard mix solution (20 μL) after the sample clean-up versus a neat standard. To evaluate the sodium and chlorine influence among the general matrix effect, saline (NaCl) solution in three different concentrations (6.0, 7.5 and 9.0 g/L milliQ water) spiked with standard mix solution were used as controls. A schematic workflow of the matrix effect experiment is given in Figure S1. The reported matrix effect values are an average of three replicates obtained from independent sample preparations within each sample group. Matrix effect was calculated according to Equation (2) if metabolite was detected with a basal level in plasma, otherwise using Equation (1).

ME = (Peak area of analyte in spiked plasma matrix)/(Peak area of analyte in neat standard solution)(1)

ME = ((Peak area of analyte in spiked plasma matrix-Peak area of analyte in unspiked plasma matrix))/(Peak area of analyte in neat standard solution)(2)

Matrix effect in the range of ±20% (0.80 < ME < 1.20) were considered as negligible and labelled as “no matrix effect”, since it is the common variability accepted in bioanalysis [13]. ME values below 0.80 were considered as ion suppression, and those above 1.20 as ion enhancement.

### 3.6. Repeatability

To evaluate the repeatability, intra- and inter-batch analysis were performed at optimal chromatography condition of each column. Therefore, ZIC-c and BEH-amide were operated at pH 7 and 10, respectively, coupled with negative ESI-MS. Prior to batch analysis, the column was equilibrated with the initial mobile phase gradient and subsequently conditioned with 20 pooled lab QC plasma injections. The intra-batch analysis included consecutive injection of 200 plasma QC samples. The inter-batch analysis included three batches, comprising injections of 20 plasma QC samples per batch with an interval of two days in between. The inter- and intra-batch variation were assessed by calculating the relative standard deviation. 

### 3.7. Data Analysis

The identification and integration of the analytes from LC-MS raw data was performed using AB Sciex PeakView™ 2.0 and MultiQuant™ 3.0.1. Related chromatographic peak parameters including retention time, peak height, peak width, tailing factor were also calculated by MultiQuant automatically. Predicted logD, charge state and hydrogen bond number under pH 3, 7, 10 for each analyte were calculated using Marvin Sketch software version 20.10.0, ChemAxon (http://www.chemaxon.com (accessed on 19 August 2020)) and shown in Table S3. To achieve good comparison between different chromatography conditions over all analytes, a chromatographic performance scoring system was defined to assign a score for each individual metabolite [14,21]. The scoring system was defined based on following parameters: metabolite retention, peak sensitivity (as indicated by natural log of the signal-to-noise ratio), peak sharpness (as indicated by natural log of the peak height), and peak symmetry (as indicated by the tailing factor). Details of scoring parameters are presented in the supporting information Table S4. The total score for each compound was calculated according to the formula (3).

Score_total = Score_retention + Score_sharpness + Score_symmerty + Score_sensitivity(3)

A simple linear regression model was used to analyse the relationship between metabolites polarity at different pH conditions and elution order. The model was built in R (version 3.6.2), and plotted a fitting line across all data points. Based on the linear model, the Pearson’s correlation coefficient (r) was calculated. For untargeted analysis, the raw data sets were converted to mzXML files and subsequently centroided using Proteo Wizard MSConvert version 3.0 [21]. Metabolite features were extracted from converted data sets using XCMS package (version 3.10) in R (version 3.6.2) [28]. Data were processed as a multi-group experiment and the parameter settings were as follows: centWave algorithm for feature detection (Δ*m*/*z* = 5 ppm, minimum peak width = 5 s and maximum peak width = 100 s, S/N threshold = 10, mzdiff = 0.01, integration method = 1, prefilter peaks = 3, prefilter intensity = 100, noise filter = 100); obiwarp settings for retention time correction (profStep = 1); and parameters for chromatogram alignment, including mzwid = 0.01, minfrac = 0.5 and bw = 5. The resulting XCMS peak table was further processed to preserve only peaks that were not present in technical blanks while being present in at least one plasma phenotype. For recognition and removal of erroneous features in the datasets, MS-FLO (http://msflo.fiehnlab.ucdavis.edu (accessed on 8 April 2021)) was used [29]. To obtain an overview of the metabolic data, abundance profiles of metabolites were glog (generalized logarithm) transformed and subjected to principal component analysis (PCA) using MetaboAnalyst version 5.0 (https://www.metaboanalyst.ca (accessed on 8 April 2021)) [30]. The retention factor (K) was calculated according to formula (4), where tR is the retention time of the feature in minutes and the t0 is the void volume of the column in minutes.

K = tR − t0/t0(4)

## 4. Conclusions

The performance of two widely used HILIC columns, BEH-amide and ZIC-c HILIC, for global metabolomics were systematically evaluated using metabolite standard solutions and human plasma samples. Mobile phase pH is the most critical parameter since it affects metabolite charge and its retention behaviour, thereby impacting the column selectivity and the quantity of detected metabolic features in untargeted analysis. Our data indicate the highest performance for ZIC-c when operated at pH 7 and for BEH-amide at pH 10. The ZIC-c HILIC column exhibited several advantages, including superior performance for different classes of compounds, better isomer separation, high repeatability and high metabolic coverage. Regardless of the column type, the retention of inorganic ions in plasma leads to extensive adduct formation and co-elution with analytes and, as a result, ion-suppression particularly for amino acids and amine classes on ZIC-c HILIC, while it acts as minor contributing factor to the overall plasma matrix effect. Further investigation on depletion of these ions during sample preparation while maintaining a high metabolic coverage is needed. Our evaluation proved high repeatability in retention time and detected peak area is achieved with the complex plasma matrix. The repeatability test also indicates that adequate equilibration and conditioning of both ZIC-c and BEH-amide columns is required prior to batch analysis, which is essential for the interpretation of untargeted data. Apart from these specific results, this work provides guidance on systematically evaluating a chromatography column performance for global plasma metabolomics studies, which is highly valuable in the future evaluation of (novel) HILIC stationary phases applied to other types of matrices. 

## Figures and Tables

**Table 1:** Summary of plasma matrix effect and sodium chloride effect on representative metabolite classes.

	ZIC-c (51 Metabolites Detected)		BEH (47 Metabolites Detected)	
Metabolite Classes	Matrix Effect	Salt Effect	Matrix Effect	Salt Effect
Amino acids	11 (16 *)	11 (16)	14 (17)	4 (17)
Amines	2 (4)	3 (4)	2 (4)	1 (4)
Sugar and Sugar phosphate	5 (5)	4 (5)	4 (4)	2 (4)
Nucleoside and Nucleotide	10 (13)	10 (13)	8 (11)	7 (11)
Acylcarnitines	1 (2)	0 (2)	0 (2)	1 (2)
Organic acids	8 (11)	8 (11)	6 (9)	6 (9)

* Numbers within brackets represent the total metabolite number for each class. Numbers before brackets represent the number of affected metabolites. For metabolites that were measured in both positive and negative modes, matrix/salt effect is marked when either mode showed ion suppression/enhancement.

## Data Availability

The data presented in this study are accessible through EBI Metabolights [31] repository accession number MTBLS4157 (www.ebi.ac.uk/metabolights/MTBLS4157 (access date on 21 January 2022)).

## Supplementary Materials

The following supporting information can be downloaded at: www.mdpi.com/article/10.3390/metabo12020165/s1, Figure S1: A schematic workflow of the matrix effect evaluation, Figure S2: The major elution profile of sodium as a sodium formate cluster on the ZIC-c HILIC column and BEH-amide HILIC column, Figure S3: Extracted ion chromatograms of metabolites with a high RSD deviation (above 20%) in peak area repeatability evaluation, Figure S4: Extracted ion chromatograms of 16 metabolites on the ZIC-c HILIC column, Figure S5: Extracted ion chromatograms of 16 metabolites on the BEH-amide column, Figure S6: The total ion chromatogram (TIC)s of each plasma sample from four different phenotypes analyzed using the untargeted HILIC-MS method, Figure S7: Bad feature showcase from untargeted plasma analysis, Table S1: List of chemical standards and stable isotope-labeled internal standards used in the study, Table S2: Columns and mobile phase conditions for ZIC-c HILIC and BEH Amide column, Table S3: Physical and chemical properties of polar target compound under pH = 3, 7, 10, Table S4: Scoring criteria for the evaluation of compound chromatography performance, Table S5: Plasma overall matrix effect and particular sodium chloride effect on metabolites analyzed using ZIC-c HILIC column, Table S6: Plasma overall matrix effect and particular sodium chloride effect on metabolites analyzed using BEH-amide HILIC, Table S7: The ratio of the (de)protonated and adduct species peak area in plasma matrix and pure sodium chloride solution compared to neat solution, Table S8: Intra- and inter-batch repeatability analysis of peak retention time and peak area, Table S9: Process of untargeted feature filtering.
