# Supplementary material for: Systematic Evaluation of HILIC Stationary Phases for Global Metabolomics of Human Plasma
Source: Metabolites. 2022 Feb 9;12(2):165. doi: 10.3390/metabo12020165 (PMC8875576; doi:10.3390/metabo12020165)
Supplement: Supplementary file 1 [file metabolites-12-00165-s001.zip › Supporting information_version2.pdf]

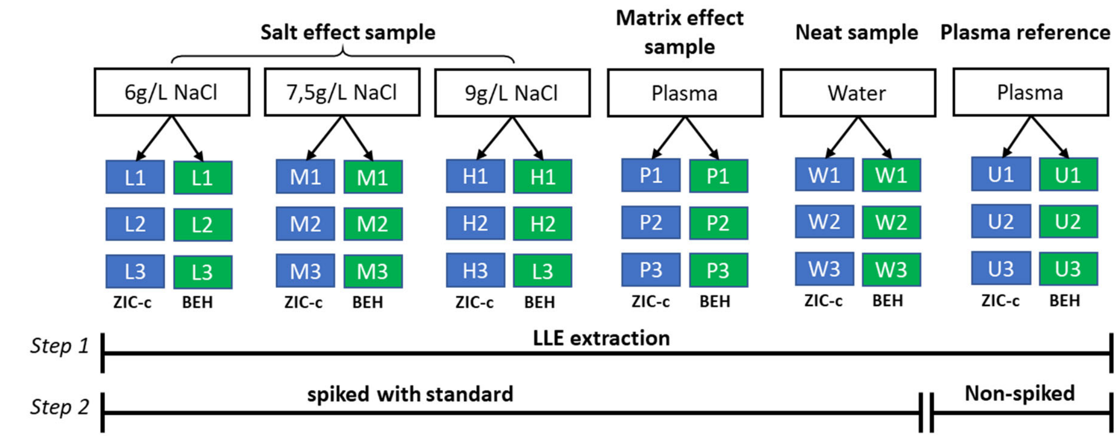

Figure S1. A schematic workflow of the matrix effect evaluation. Each group has three replicates processed independently. L: low concentration; M: medium concentration; H: high concentration; P: plasma spiked with standard; W: water spiked with standard; U: non-spiked plasma.

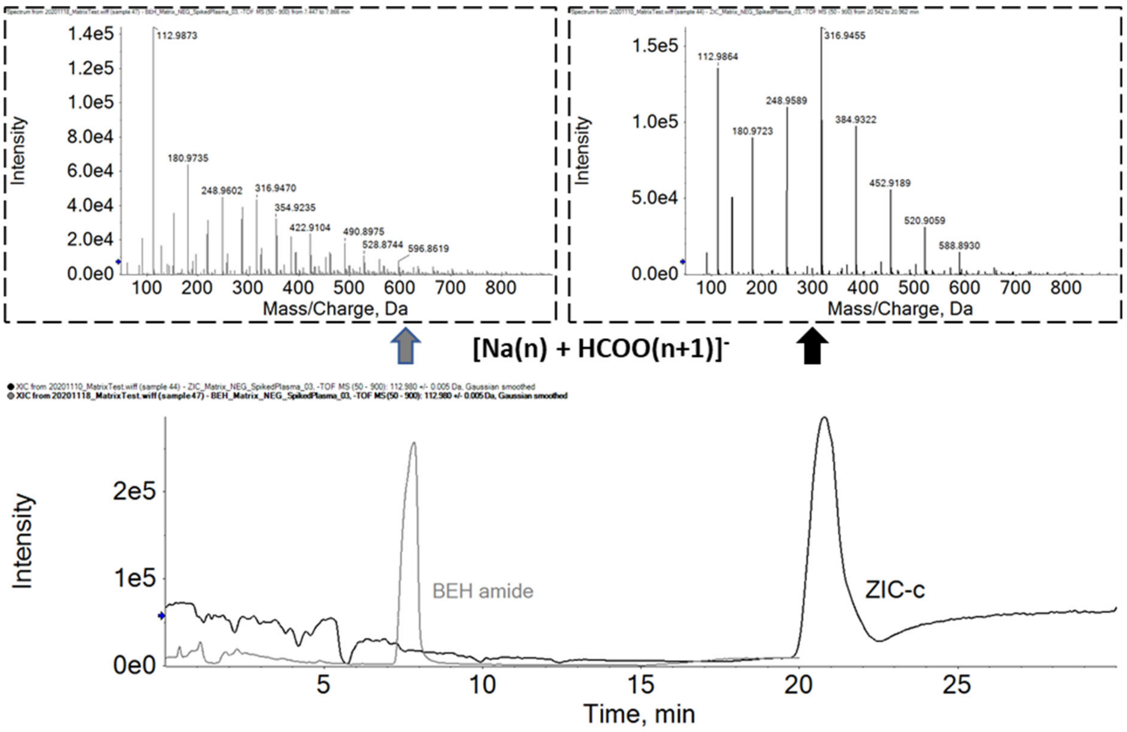

Figure S2. The major elution profile of sodium as a sodium formate cluster on the ZIC-c HILIC column and BEH Amide HILIC column. The extracted ion chromatograms of [Na(n) + HCOO(n+1)]- are shown below. The corresponding mass spectra at the elution time range are shown above.

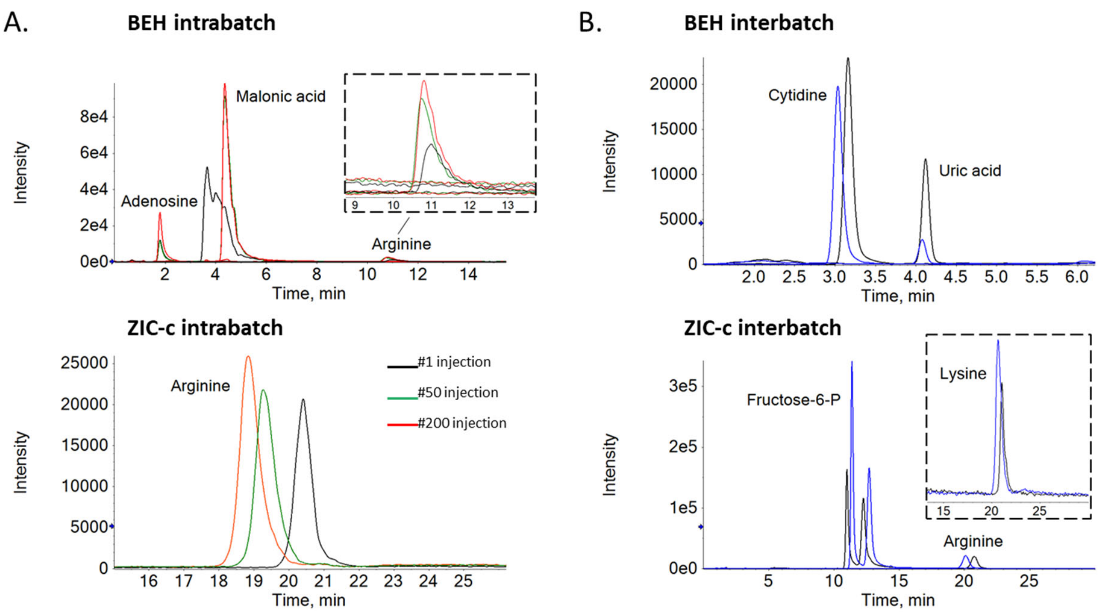

Figure S3. Extracted ion chromatograms of metabolites with a high RSD deviation (above 20%) in peak area reproducibility evaluation.

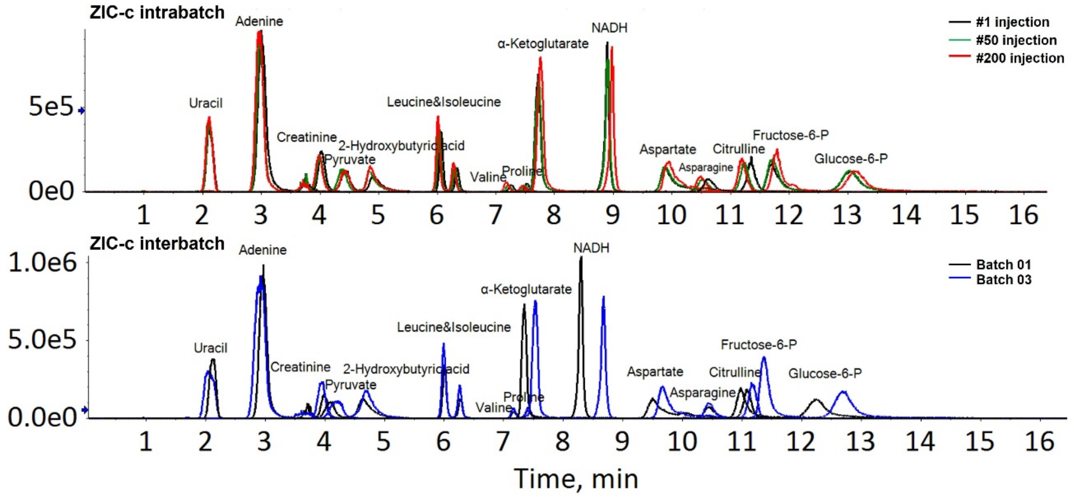

Figure S4. Extracted ion chromatograms of 16 metabolites covering the amino acid, amine, sugar phosphate, nucleoside, nucleotide, and organic acid classes presented for repeatability evaluation of retention time and peak area on the ZIC-c HILIC column.

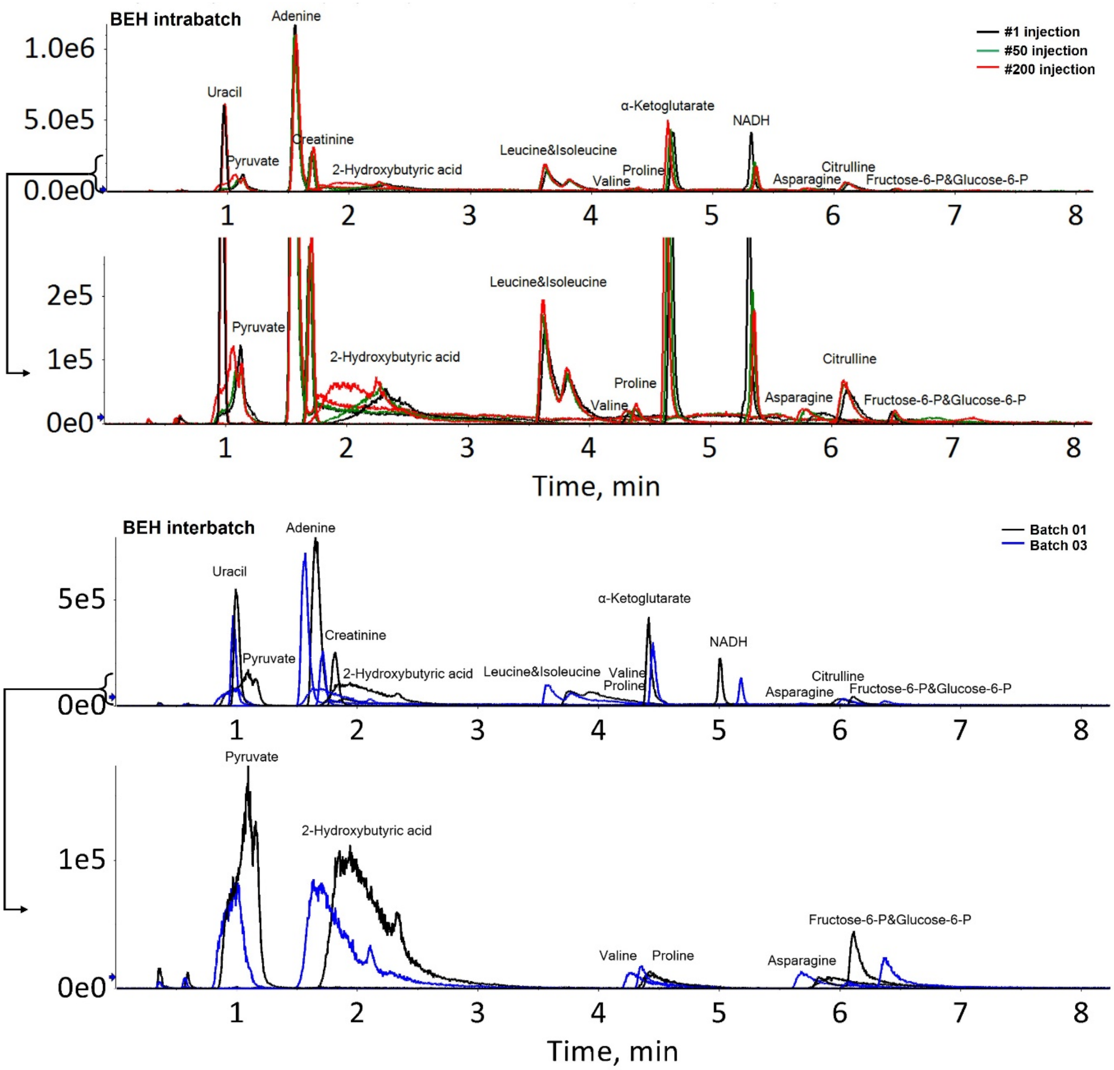

Figure S5. Extracted ion chromatograms of 15 metabolites covering the amino acid, amine, sugar phosphate, nucleoside, nucleotide, and organic acid classes presented for repeatability evaluation of retention time and peak area on the BEH-amide HILIC column.

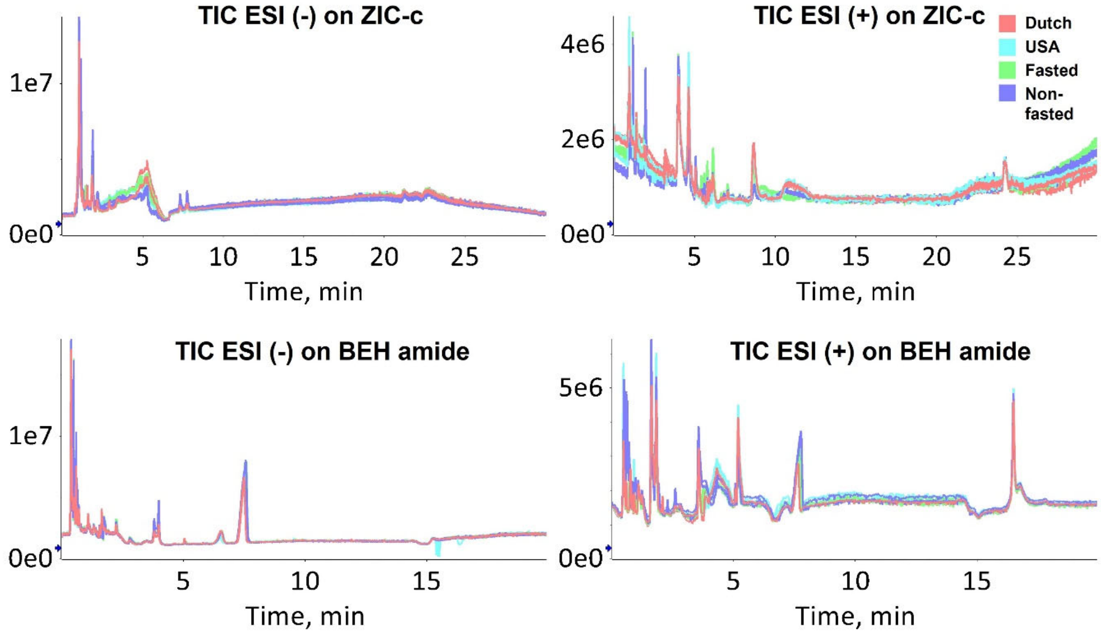

Figure S6. The total ion chromatogram (TIC)s of each plasma sample from four different phenotypes analyzed using the untargeted HILIC-MS method. Each phenotype has three replicates, extracted and measured independently. (Red for Dutch, Blue for USA, Green for fasted, Purple for non-fasted)

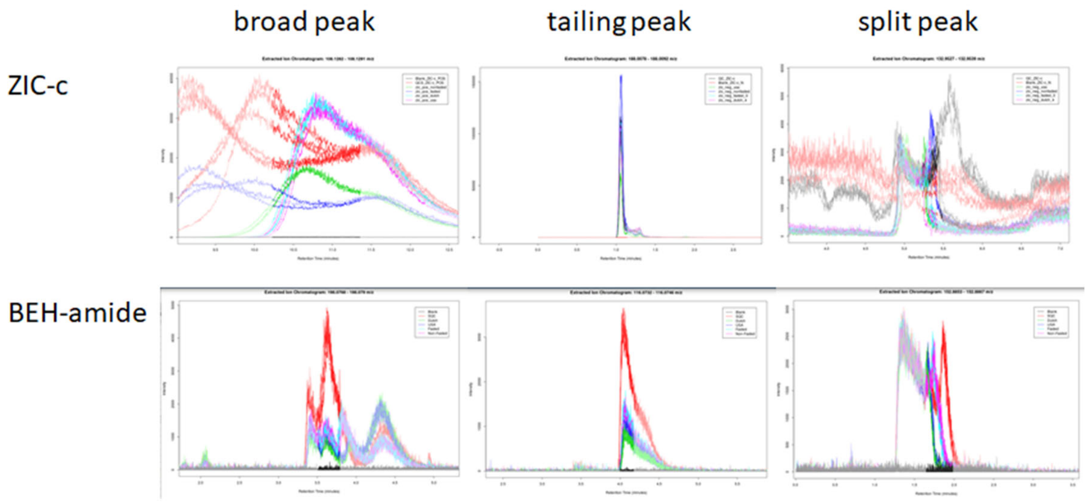

Figure S7. Bad feature showcase from untargeted plasma analysis.
